# Prognostic analysis of the plasma fibrinogen combined with neutrophil‐to‐lymphocyte ratio in patients with non–small cell lung cancer after radical resection

**DOI:** 10.1111/1759-7714.14883

**Published:** 2023-04-10

**Authors:** Gao‐Xiang Wang, Zhi‐Ning Huang, Ying‐Quan Ye, Shan‐Ming Tao, Mei‐Qing Xu, Mei Zhang, Ming‐Ran Xie

**Affiliations:** ^1^ Department of Chinese Integrative Medicine Oncology The First Affiliated Hospital of Anhui Medical University Hefei China; ^2^ Department of Integrated Traditional Chinese and Western Medicine Anhui Medical University Hefei China; ^3^ Department of Thoracic Surgery The First Affiliated Hospital of USTC Hefei China; ^4^ Division of Life Sciences and Medicine University of Science and Technology of China Hefei China

**Keywords:** neutrophil‐to‐lymphocyte ratio (NLR), non–small cell lung cancer (NSCLC), surgery, survival

## Abstract

**Background:**

To investigate the correlation between the fibrinogen combined with neutrophil‐to‐lymphocyte ratio (F‐NLR) and the clinicopathologic features of non–small cell lung cancer (NSCLC) patients who underwent radical resection.

**Methods:**

This study reviewed the medical records of 289 patients with NSCLC who underwent radical resection. The patients were stratified into three groups based on F‐NLR as follows: patients with low NLR and fibrinogen were group A, patients with high NLR or fibrinogen were group B, and patients with high NLR and fibrinogen were group C. Receiver operating characteristic curve and Youden index were used to determine the cutoff value of the NLR and fibrinogen. Survival curves were described by Kaplan–Meier method and compared by log‐rank test. The univariate and multivariate analyses were performed with the Cox proportional hazard model to identify the prognostic factors.

**Results:**

A value of 3.19 was taken as the optimal cutoff value of NLR in this study. A value of 309 was used as the optimal cutoff value of fibrinogen. Cox multivariate analysis showed that tumor, nodes, metastasis (TNM) stage and F‐NLR were independent prognostic factors affecting the survival rate of patients. The first‐, third‐, and fifth‐year survival rates in group A were 99.2%, 96.6%, and 95.0%, respectively. The first‐, third‐, and fifth‐year survival rates in group B were 98.4%, 76.6%, and 63.2%, respectively. The first‐, third‐, and fifth‐year survival rates in group C were 91.3%, 41.1%, and 22.8%, respectively. F‐NLR was significantly correlated with overall survival in patients with NSCLC (*p* < 0.001).

**Conclusions:**

The F‐NLR level is markedly related to the prognosis of patients with NSCLC undergoing radical surgery. Therefore, closer attention should be given to patients with NSCLC with a high F‐NLR before surgery to provide postoperative adjuvant therapy.

## Graphical abstract

The optimal cutoff values of neutrophil‐to‐lymphocyte ratio and fibrinogen were found by receiver operating characteristic curve, and a new prediction index fibrinogen combined with neutrophil‐to‐lymphocyte ratio (F‐NLR) was constructed. The F‐NLR level is markedly related to the prognosis of patients with non–small cell lung cancer (NSCLC) undergoing radical surgery. The long‐term survival rate of patients with a high F‐NLR before surgery is lower than that of patients with a low F‐NLR before surgery. Therefore, closer attention should be given to patients with NSCLC with a high F‐NLR before surgery to provide timely intervention in tumor recurrence and metastasis and obtain a better prognosis.

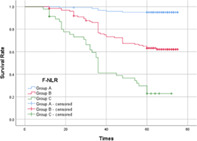

## INTRODUCTION

Lung cancer mortality has always been the highest rate among those of all the malignant tumors in the world.^1^, ^2^ The histological types of lung cancer include small cell lung cancer (SCLC) and non–small cell lung cancer (NSCLC), of which NSCLC accounts for 80% to 85%.^3^, ^4^ Surgery is the main treatment for resectable NSCLC, but single surgery is not sufficient for most lung cancer patients and requires a combination of adjuvant treatment.^5^, ^6^ Therefore, the question of identifying high‐risk patients with postoperative recurrence and metastasis in advance and providing appropriate clinical intervention is a hot topic in academic research. Tumor, node, and metastasis (TNM) stage is the most commonly used prognostic indicator regarding lung cancer in clinical practice, but patients with the same TNM stage do not have the same prognosis. In addition, some biomarkers related to the prognosis of NSCLC have been identified. Among them, carcinoembryonic antigen (CEA) is the most common.^7^, ^8^, ^9^ However, its sensitivity and specificity for prognosis are not sufficient. In recent years, an increasing number of biological indicators and mathematical models have been used to predict the prognosis of lung cancer patients and guide clinical treatment. However, tests based on biological indicators are often expensive and cannot be widely used as routine approaches.

In recent decades, the systemic inflammatory response has been associated with cancer progression.^10^, ^11^ Previous studies have confirmed the effectiveness of inflammatory markers as prognostic indicators. The neutrophil‐lymphocyte ratio (NLR) reflects the host's inflammatory and immune response status. It has been reported in the literature that the NLR has a substantial impact on the prognosis of many malignant tumors, such as NSCLC, gastric cancer, colorectal cancer, and pancreatic cancer.^12^, ^13^, ^14^, ^15^ Higher NLR values predict poor prognosis. Correspondingly, coagulation and fibrinolytic system markers, including fibrinogen levels, are associated with tumor progression in different types of malignant tumors.^16^, ^17^ In recent years, studies have shown that the combination of fibrinogen level and values of NLR can be used to predict the survival of patients with esophageal cancer, gastric cancer, and colorectal cancer.^18^, ^19^, ^20^ However, few studies have reported the combined application of the NLR and fibrinogen level (F‐NLR) in patients with NSCLC. This study involved enrollment of a total of 289 patients with NSCLC who were pathologically diagnosed in the Department of Thoracic Surgery of the First Affiliated Hospital of the University of Science and Technology of China between June 2016 and June 2017 and compared and analyzed the effectiveness of the F‐NLR score in evaluating the long‐term survival of patients with NSCLC after radical resection. The results are as follows.

## METHODS

### Participants and study design

This study involved enrollment of 568 patients with NSCLC who underwent radical resection of lung cancer between June 2016 and June 2017 in the Department of Thoracic Surgery of the First Affiliated Hospital of the University of Science and Technology of China. The case inclusion criteria were as follows: (1) NSCLC confirmed by pathology after operation; (2) received systematic mediastinal lymph node dissection; (3) underwent lobectomy or pneumonectomy, and R0 resection. Exclusion criteria were as follows: (1) received neoadjuvant treatment before surgery; (2) underwent sublobectomy or selective lymph node dissection; (3) and incomplete case data.

Based on the above criteria, a total of 289 patients were included in this study. There were 173 males and 116 females; there were 211 patients in stage I, 42 patients in stage II and 36 patients in stage III.

Pretreatment examinations included chest and upper abdominal computed tomography (CT) enhancement, craniocerebral magnetic resonance (plain scan + enhancement), bone scan, electronic bronchoscope, electrocardiogram, and lung function testing. Patients older than 65 years old underwent echocardiography. A preoperation test was routine. This retrospective study was approved by the First Affiliated Hospital of China University of Science and Technology (2022‐RE‐442).

### F‐NLR

Blood was drawn from all patients for routine blood and biochemical examinations within 1 week before the operation. According to the results, the NLR value was calculated. The calculation formula was NLR = peripheral blood neutrophil count (×10^9^/L)/peripheral blood lymphocyte count (×10^9^/L). The optimal cutoff values of NLR and plasma fibrinogen were determined by receiver operating characteristic (ROC) curve analysis. The patients were stratified into three groups based on F‐NLR as follows: patients with low NLR and fibrinogen were group A, patients with high NLR or fibrinogen were group B, and patients with high NLR and fibrinogen were group C.

### Measures

The initial hospitalization during the years under study was identified as the index visit. Patient characteristics and clinical laboratory data during the index visit were extracted from hospital records, including the patient's sex, age, smoking history, histological classification, degree of differentiation, and TNM stage. TNM staging was based on the International Association for Lung Cancer Research guidelines, 8th edition. The overall survival (OS) was defined as the time period from the date of the operation for NSCLC until death from any cause.

### Statistical analysis

Follow‐up was carried out in two ways: regular outpatient follow‐up and telephone follow‐up. Follow‐up was conducted once every 3 months in the first year after the operation, once every 6 months in the second year, and once every year from the third year to obtain relevant clinical information (including chest, brain CT, bone scan, abdominal and adrenal ultrasound, etc.) and patient survival data.

**FIGURE 1:**
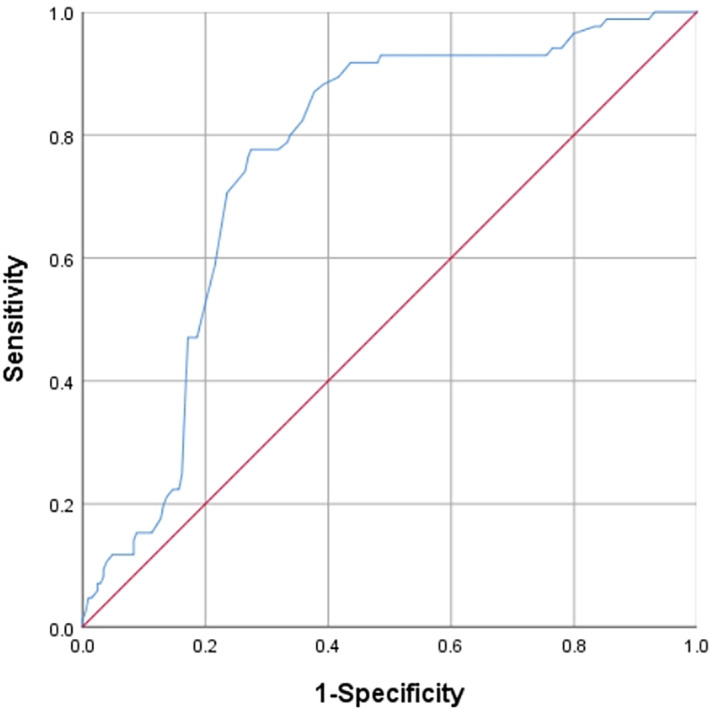
Receiver operating curve analysis for the optimal cutoff value of neutrophil‐to‐lymphocyte ratio.

**FIGURE 2:**
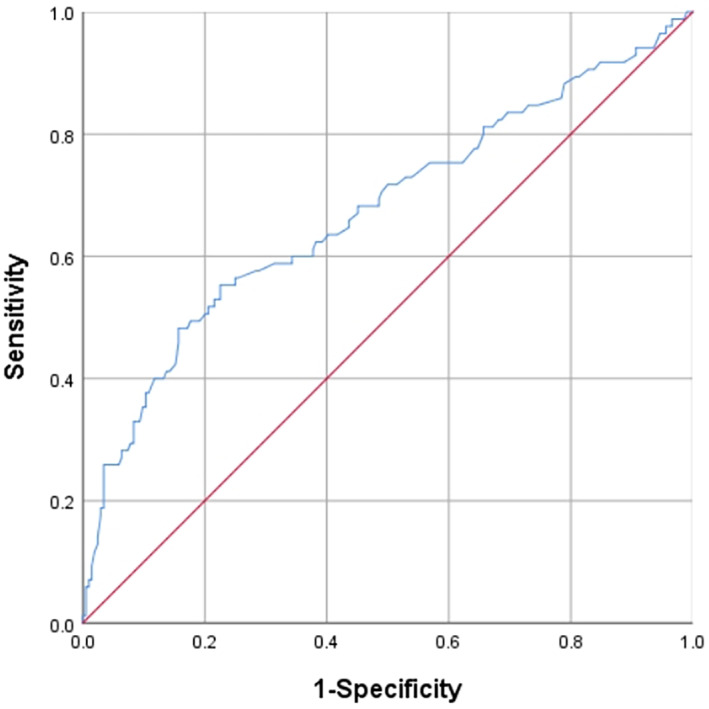
Receiver operating curve analysis for the optimal cutoff value of fibrinogen.

SPSS 26.0 statistical software was used for data analysis. The sensitivity and specificity of the 5‐year total survival time were evaluated by drawing the working characteristic curve of the subjects, and the optimal cutoff value of NLR and fibrinogen was determined by calculating the Youden index (Figures 1 and 2). For quantitative variables, the *t* test was used to evaluate normally distributed data. Non‐normally distributed data were analyzed with the Mann–Whitney test. Qualitative variables were examined with Pearson's χ^2^ test when appropriate. Data are expressed as the median and interquartile range. Survival curves for the two groups were estimated using the Kaplan–Meier method and compared by a log‐rank test. *p* < 0.05 was considered indicative of statistical significance.

## RESULTS

### Determination of critical values of NLR and fibrinogen

The ROC of NLR and fibrinogen was plotted using the 5‐year survival rate as the endpoint (Figures 1 and 2), and the optimal cutoff value was determined by calculating the Youden index [sensitivity − (1 − specificity)]. The area under the ROC of the NLR was 0.760. When the NLR value was 3.19, the sensitivity was 77.6%, the specificity was 72.5%, and the maximum Youden index was 0.501. A value of 3.19 was taken as the optimal cutoff value of NLR in this study. The ROC area of fibrinogen was 0.674. When the fibrinogen value was 309, the sensitivity was 55.3%, the specificity was 77.5%, and the Youden index was 0.328. A value of 309 was used as the optimal cutoff value of fibrinogen.

When NLR was <3.19 and fibrinogen was <309, the patients were in group A. A total of 120 cases; when NLR was >3.19 or fibrinogen was >309, the patients were in group B. A total of 123 cases; and when NLR was >3.19 and fibrinogen was >309, the patients were in group C. A total of 46 cases.

### Association between NLR and fibrinogen level and clinicopathological factors

Table 1 shows the association between clinicopathological factors and NLR and fibrinogen level. Compared with a low NLR, a high NLR was associated with age (*p* = 0.038) and TNM stage (*p* < 0.001). Compared with a low fibrinogen level, a high fibrinogen level was correlated with heavy smoking history (*p* = 0.011), squamous cell carcinoma (*p* = 0.018) and high stage III (*p* < 0.001). Therefore, a high fibrinogen level was significantly associated with advanced‐stage diseases.

**TABLE 1:** Clinical characteristics according to NLR and fibrinogen

Index	NLR		χ^2^	*p*	Fibrinogen		χ^2^	*p*
	Low	High			Low	High		
Sex			1.494	0.222			2.643	0.104
Male	105	68			111	62		
Female	62	54			85	31		
Age, years			4.325	0.038			1.062	0.303
≤60	75	40			82	33		
>60	92	82			114	60		
Smoking history			1.203	0.273			6.462	0.011
Yes	36	20			30	26		
No	131	102			166	67		
Histological classification			3.969	0.137			7.999	0.018
Adenocarcinoma	122	92			154	60		
Squamous cell carcinoma	32	27			31	28		
Others	13	3			11	5		
Degree of differentiation			0.294	0.863			0.429	0.807
Low	122	90			142	70		
Middle	24	19			31	12		
High	21	13			23	11		
Postoperative adjuvant therapy			2.105	0.147			0.174	0.676
Yes	27	28			36	19		
No	140	94			160	74		
TNM stage			25.549	<0.001			28.130	<0.001
I	136	75			161	50		
II	24	18			22	20		
III	7	29			13	23		

Abbreviations: NLR, neutrophil‐to‐lymphocyte ratio; TNM, Tumor, node, and metastasis.

### Prognostic importance of NLR and fibrinogen level

**FIGURE 3:**
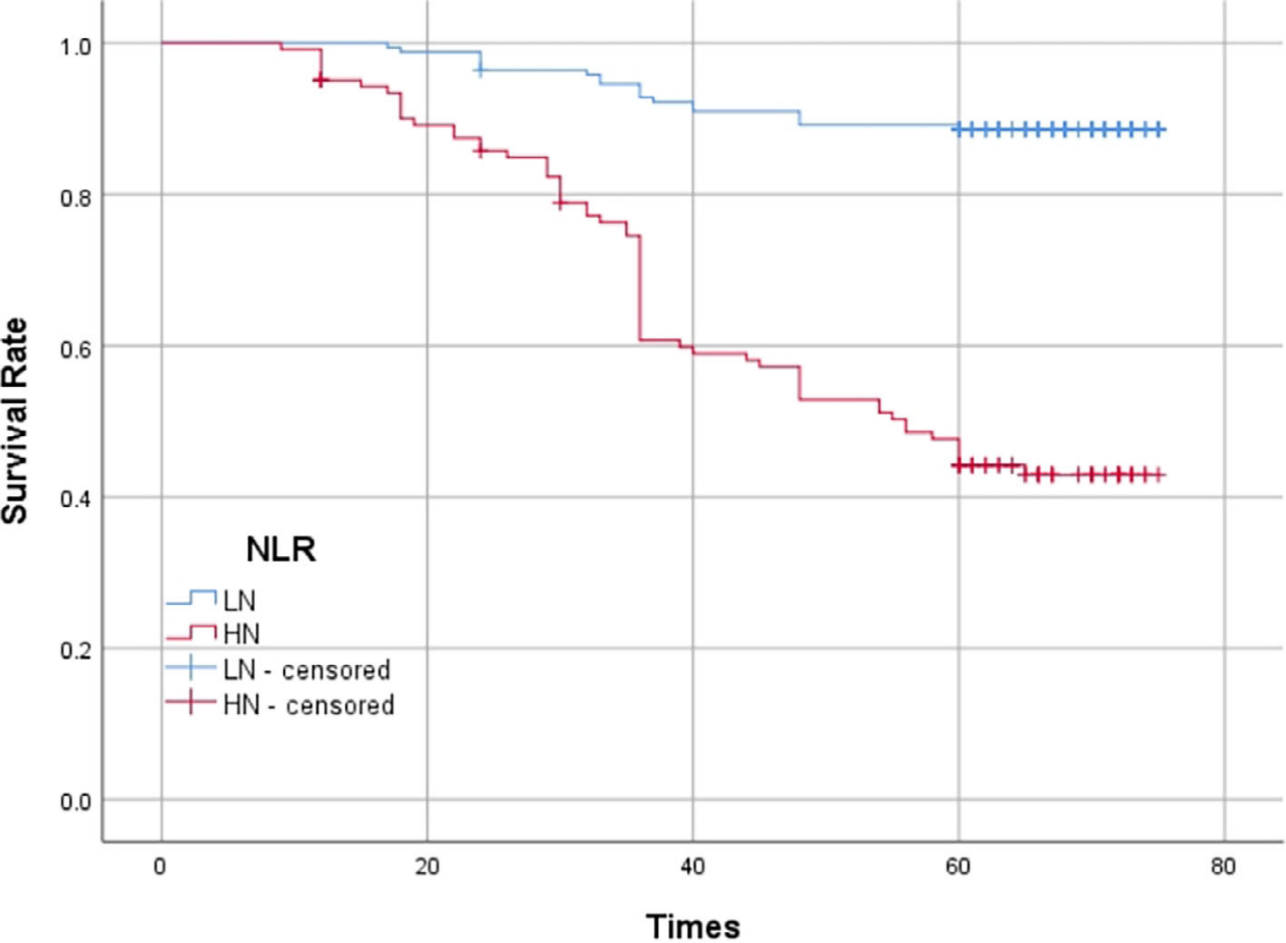
Kaplan–Meier curves of survival rate according to neutrophil‐to‐lymphocyte ratio.

**FIGURE 4:**
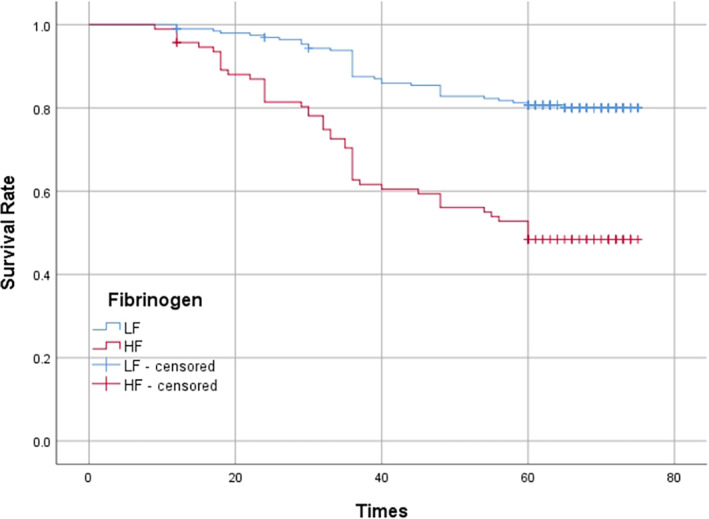
Kaplan–Meier curves of survival rate according to fibrinogen.

Of the 289 patients were included in single and multiple factor analyses. The follow‐up time ranged from June 2016 to October 2022, with a total follow‐up time of 76.0 months and a median follow‐up time of 68.0 months. The first‐, third‐, and fifth‐year survival rates for the whole group were 96.2%, 79.2%, and 70.6%, respectively. In the high NLR group, the first‐, third‐, and fifth‐year survival rates were 95.1%, 60.7%, and 44.2%, respectively. In the low NLR group, the first‐, third‐, and fifth‐year survival rates were 99.4%, 92.8%, and 88.6%, respectively. The survival rate of patient in the low NLR group was significantly better than that of patients in the high NLR group (*p* < 0.001) (Figure 3). In the high fibrinogen group, the first‐, third‐, and fifth‐year survival rates were 95.7%, 62.7%, and 48.4%, respectively. In the low fibrinogen group, the first‐, third‐, and fifth‐year survival rates were 99.0%, 87.5%, and 80.7%, respectively. The survival rate of patient in the low fibrinogen group was significantly better than that of patients in the high fibrinogen group (*p* < 0.001) (Figure 4).

### Relationship between F‐NLR and clinicopathological characteristics of patients

The three groups were similar in terms of age, sex, smoking history, histological classification, degree of differentiation, and postoperative adjuvant therapy, there were no significant differences (*p* > 0.05) (Table 2). A high F‐NLR was associated with high TNM stage (*p* < 0.001).

**TABLE 2:** Clinical characteristics according to F‐NLR

Index	Group A	Group B	Group C	χ^2^	*p*
Sex				2.321	0.313
Male	74	68	31		
Female	46	55	15		
Age, years				4.965	0.084
≤60	54	49	12		
>60	66	74	34		
Smoking history				0.973	0.615
Yes	20	26	10		
No	100	97	36		
Histological classification				7.705	0.103
Adenocarcinoma	92	92	30		
Squamous cell carcinoma	18	27	14		
Others	10	4	2		
Degree of differentiation				1.428	0.839
Low	85	94	33		
Middle	20	15	8		
High	15	14	5		
Postoperative adjuvant therapy				4.945	0.084
Yes	22	19	14		
No	98	104	32		
TNM stage				53.626	<0.001
I	103	91	17		
II	14	18	10		
III	3	14	19		

Abbreviations: F‐NLR, fibrinogen combined with neutrophil‐to‐lymphocyte ratio; TNM, Tumor, node, and metastasis.

### Prognostic analysis based on F‐NLR among patients with NSCLC

**FIGURE 5:**
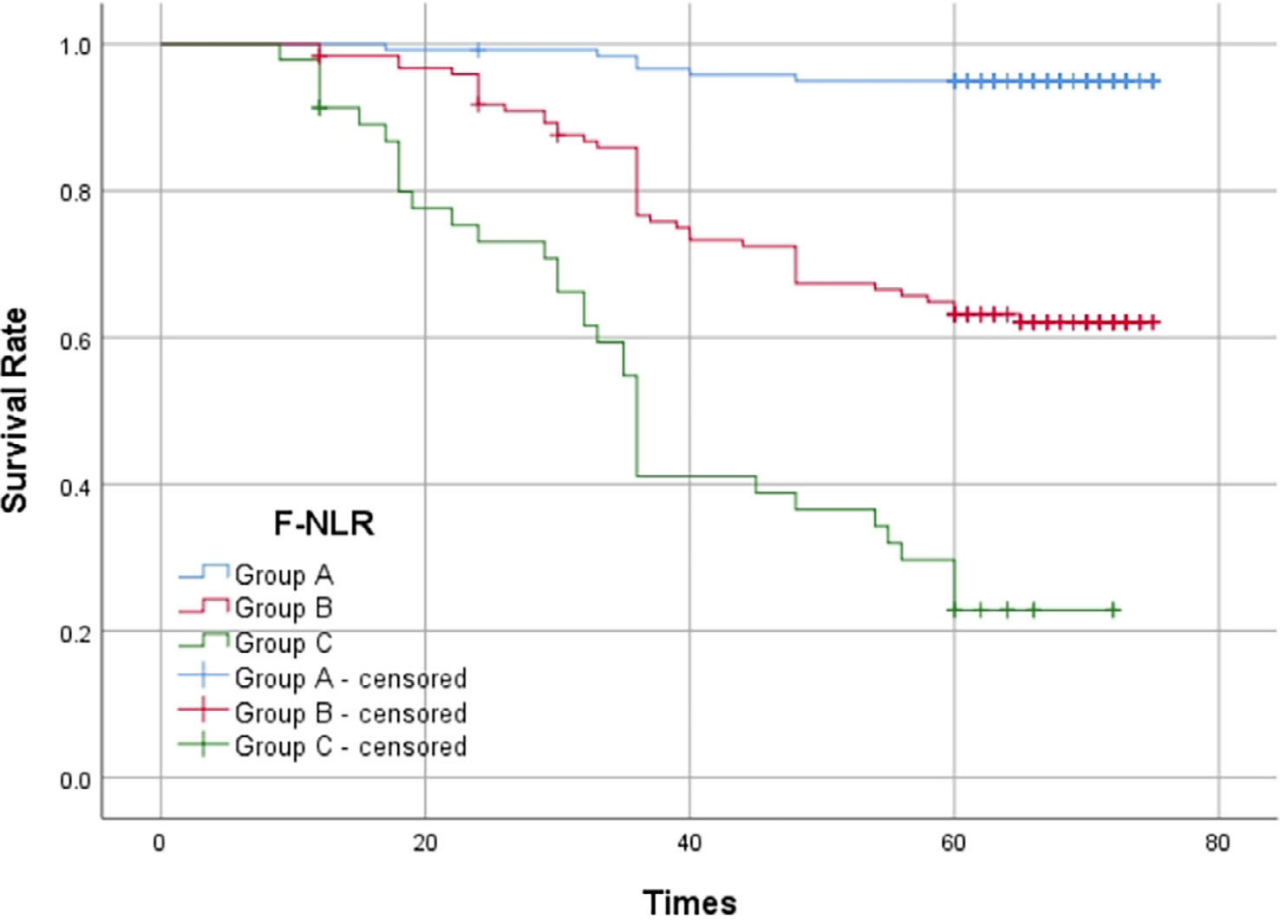
Kaplan–Meier curves of survival rate according to fibrinogen combined with neutrophil‐to‐lymphocyte ratio.

F‐NLR was significantly correlated with OS in patients with NSCLC (*p* < 0.001) (Figure 5). The first‐, third‐, and fifth‐year survival rates in group A were 99.2%, 96.6%, and 95.0%, respectively. The first‐, third‐, and fifth‐year survival rates in group B were 98.4%, 76.6%, and 63.2%, respectively. The first‐, third‐, and fifth‐year survival rates in group C were 91.3%, 41.1%, and 22.8%, respectively. The survival rate of patients in group A was significantly better than that of patients in group B and group C (*p* < 0.001).

### Univariate and multivariate analyses of OS

The univariate analysis of the clinicopathological data of patients showed that TNM stage, NLR, fibrinogen, and F‐NLR were significantly correlated with the survival rate of patients (*p* < 0.05) (Table 3). The clinical and pathological data of patients were substituted into the Cox model for multifactor analysis. The results showed that TNM stage and F‐NLR were independent prognostic factors affecting the survival rate of patients (*p* < 0.05, Table 4).

**TABLE 3:** Univariate analysis of prognostic factors influencing survival rate

Index	Cases	5‐year survival rate (%)	*p*
Sex			0.755
Male	173	69.8	
Female	116	71.1	
Age, years			0.065
≤60	115	76.2	
>60	174	66.4	
Smoking history			0.906
Yes	56	70.7	
No	233	68.6	
Histological classification			0.160
Adenocarcinoma	214	71.7	
Squamous cell carcinoma	59	62.3	
Others	16	75.0	
Degree of differentiation			0.087
Low	212	69.7	
Middle	43	61.2	
High	34	85.3	
Postoperative adjuvant therapy			0.492
Yes	55	66.2	
No	234	71.3	
TNM stage			<0.001
I	211	79.4	
II	42	66.7	
III	36	16.4	
NLR			<0.001
Low	167	88.6	
High	122	44.2	
Fibrinogen			<0.001
Low	196	80.7	
High	93	48.4	
F‐NLR			<0.001
Group A	120	95.0	
Group B	123	63.2	
Group C	46	22.8	

Abbreviations: TNM, Tumor, node, and metastasis; NLR, neutrophil‐to‐lymphocyte ratio; F‐NLR, fibrinogen combined with neutrophil‐to‐lymphocyte ratio.

**TABLE 4:** Multivariate analysis of prognostic factors influencing survival rate

Index	*p*	HR (95% CI)
F‐NLR	<0.001	3.375 (2.431, 4.687)
TNM stage	<0.001	2.053 (1.559, 2.703)
Sex	0.506	
Age, years	0.088	
Smoking history	0.358	
Histological classification	0.248	
Degree of differentiation	0.254	
Postoperative adjuvant therapy	0.561	

Abbreviations: HR, hazard ratio; CI, confidence interval; F‐NLR, fibrinogen combined with neutrophil‐to‐lymphocyte ratio; TNM, Tumor, node, and metastasis.

## DISCUSSION

At present, NSCLC is still the most common malignant tumor in terms of incidence. The question of how to screen high‐risk patients with recurrence and metastasis among NSCLC patients after radical surgery and implement early intervention is one of the major issues facing clinicians. In recent years, studies have shown that the systemic inflammatory response, such as reflected by NLR and FPR, is related to prognosis in various cases of solid tumors.^21^, ^22^, ^23^ However, the prediction accuracy of its single inflammatory index is relatively poor. Therefore, recent studies have combined fibrinogen level and NLR to predict the survival of patients with esophageal cancer, gastric cancer, and ovarian cancer.^18^, ^19^, ^24^ However, few studies have reported the combined application of plasma F‐NLR in patients with NSCLC. This study found that the preoperative F‐NLR was significantly associated with the long‐term survival rate of NSCLC patients undergoing radical resection. Multivariate prognostic analysis showed that the preoperative F‐NLR was an independent factor that could well predict the prognosis of patients.

This study found that high NLR was associated with advanced age and late pathological stage in NSCLC patients, predicting a poor prognosis. Takahashi et al.,^25^ found that high NLR was significantly correlated with patients of older age, preoperative hypoalbuminemia, and non‐adenocarcinoma histology. This study demonstrated that high fibrinogen is associated with smoking history, non‐adenocarcinoma, advanced pathology, and poor prognosis. This result was similar to that of previous research.^26^ Heavy smokers were associated with higher fibrinogen levels or higher F‐NLR scores. Cigarette smoke increases the levels of several proinflammatory cytokines and fibrinogen as a general inflammatory response to persistent stimulation, supporting the present results.^27^, ^28^ In addition, previous studies found that F‐NLR was associated with the depth of tumor invasion, lymph node metastasis, and late pathological stage in patients with esophageal and gastric cancer.^29^, ^30^ Patients with a high F‐NLR predicted a poor prognosis for patients with a lower F‐NLR.^31^ Therefore, F‐NLR is a combination of fibrinogen level and NLR and is a more effective prognostic stratification indicator.

Systemic inflammatory reactions can affect the immune response of patients and cause tumor progression. Neutrophils are an important component of tumor inflammation and immunity. Fibrinogen is a dimer synthesized by the liver, an important component of the blood coagulation reaction, and a major acute phase response protein of chronic inflammation. The increase in fibrinogen can substantially affect the prognosis of tumors. This study found that the 5‐year survival rate of patients with a high F‐NLR before surgery were significantly lower than those of patients with a low F‐NLR. Iwasaki et al.^32^ found that a high F‐NLR was an independent poor prognostic factor for OS in NSCLC patients. Huang et al.^33^ also reached the same conclusion. We believe that the reason may be as follows: first, the increase in neutrophils can reconstruct the function of the tumor extracellular matrix, therefore, promoting tumor growth and metastasis. Second, neutrophils can inhibit the cytotoxicity of lymphocytes to tumor cells and inhibit the proliferation of T cells. Third, the unlimited proliferation and invasion of tumor cells requires consumption of a large amount of protein, while the synthesis of normal protein is also affected, resulting in malnutrition and immune decline in patients while also causing tumor cell immune escape and accelerating the progression of tumors. Therefore, the high F‐NLR in NSCLC patients means that their immune response is reduced, promoting tumor development, and an antitumor imbalance in the body.

The tumor microenvironment plays an important role in tumor occurrence and development, and the inflammatory response mediated by inflammatory cells plays an important role.^34^, ^35^ Representing the main type of white blood cell, neutrophils can not only play a role in killing tumor cells, but also stimulate the growth of tumor cells. A high neutrophil count indicates that the inflammatory reaction in the body is heavy, which can provide a favorable environment for the growth of tumor cells.^36^, ^37^ At the same time, lymphocytes play an important role in tumor immune regulation. For patients with NSCLC, a low lymphocyte count indicates poor prognosis. Therefore, the NLR index can predict the prognosis of patients with NSCLC. However, the accuracy of a single indicator is low. Fibrinogen promotes the proliferation of fibroblast growth factor‐2 and vascular endothelial growth factor. The former promotes the growth of tumor cells, whereas the latter creates a good microenvironment for cancer cells. Stimulated by inflammatory factors or tumors, activated thrombin converts fibrinogen into fibrin, forms a stable skeleton and extracellular matrix around tumor cells, and prevents natural killer cells from killing tumor cells.^38^, ^39^ Therefore, combining the plasma fibrinogen index with the NLR is a more effective prognostic stratification index.

For the selection of the critical values of NLR and fibrinogen, some studies refer to the selection of the critical values of other studies. In addition, some studies selected median and average as their critical values.^18^, ^19^, ^20^, ^32^ Patients with different tumors and different regions of involvement exhibit heterogeneity, and the combined effects have an impact on the optimal threshold. In addition, there are various factors affecting data collection, sorting, and calculation, therefore, it is not rigorous to use the median method to obtain the optimal critical value. This study evaluated the sensitivity and specificity of the 5‐year survival rate by drawing an ROC curve and determined the optimal critical values of the NLR and fibrinogen level by calculating the Youden index. The research results are more reliable.

This study has the following shortcomings: First, because this study was a single‐center retrospective analysis and bore a certain case‐selective bias. Second, the study was small in sample size, and the results will need to be confirmed with prospective research involving a larger sample. Third, there are no other factors associated with the complications associated with chronic diseases and surgery that may affect surgical outcomes and long‐term survival.

## CONCLUSIONS

In summary, the F‐NLR level is markedly related to the prognosis of patients with NSCLC undergoing radical surgery. The long‐term survival rate of patients with a high F‐NLR before surgery is lower than that of patients with a low F‐NLR before surgery. Therefore, closer attention should be given to patients with NSCLC with a high F‐NLR before surgery to provide timely intervention in tumor recurrence and metastasis and obtain a better prognosis.

## AUTHOR CONTRIBUTIONS

*Conception and design*: Gao‐Xiang Wang, Mei Zhang, and Ming‐Ran Xie. *Administrative support*: Mei Zhang and Ming‐Ran Xie. *Provision of study materials or patients*: Zhi‐Ning Huang and Mei‐Qing Xu. *Collection and assembly of data*: Ying‐Quan Ye and San‐Ming Tao. *Data analysis and interpretation*: Gao‐Xiang Wang and Ying‐Quan Ye. *Manuscript writing*: All authors. *Final approval of manuscript*: All authors.

## FUNDING INFORMATION

This work was supported by the National Natural Science Foundation of China (81973643 to M.R.X.)

## CONFLICT OF INTEREST STATEMENT

The authors report no conflict of interest.
